# Heart Failure With Midrange Ejection Fraction: Prior Left Ventricular Ejection Fraction and Prognosis

**DOI:** 10.3389/fcvm.2021.697221

**Published:** 2021-08-02

**Authors:** Xinxin Zhang, Yuxi Sun, Yanli Zhang, Feifei Chen, Shuyuan Zhang, Hongyan He, Shuang Song, Gary Tse, Ying Liu

**Affiliations:** ^1^Heart Failure and Structural Cardiology Ward, The First Affiliated Hospital of Dalian Medical University, Dalian, China; ^2^Kent and Medway Medical School, Canterbury, United Kingdom

**Keywords:** heart failure, mid-range ejection fraction, prior, left ventricular ejection fraction, prognosis

## Abstract

**Aims:** Evidence-based guidelines for heart failure management depend mainly on current left ventricular ejection fraction (LVEF). However, fewer studies have examined the impact of prior LVEF. Patients may enter the heart failure with midrange ejection fraction (HFmrEF) category when heart failure with preserved ejection fraction (HFpEF) deteriorates or heart failure with reduced ejection fraction (HFrEF) improves. In this study, we examined the association between change in LVEF and adverse outcomes.

**Methods:** HFmrEF patients with at least two or more echocardiograms 3 months apart at the First Affiliated Hospital of Dalian Medical University between September 1, 2015 and November 30, 2019 were identified. According to the prior LVEF, the subjects were divided into improved group (prior LVEF < 40%), stable group (prior LVEF between 40 and 50%), and deteriorated group (prior LVEF ≥ 50%). The primary outcomes were cardiovascular death, all-cause mortality, hospitalization for worsening heart failure, and composite event of all-cause mortality or all-cause hospitalization.

**Results:** A total of 1,168 HFmrEF patients (67.04% male, mean age 63.60 ± 12.18 years) were included. The percentages of improved, stable, and deteriorated group were 310 (26.54%), 334 (28.60%), and 524 (44.86%), respectively. After a period of follow-up, 208 patients (17.81%) died and 500 patients met the composite endpoint. The rates of all-cause mortality were 35 (11.29%), 55 (16.47%), and 118 (22.52%), and the composite outcome was 102 (32.90%), 145 (43.41%), and 253 (48.28%) for the improved, stable, and deteriorated groups, respectively. Cox regression analysis showed that the deterioration group had higher risk of cardiovascular death (HR: 1.707, 95% CI: 1.064–2.739, *P* = 0.027), all-cause death (HR 1.948, 95% CI 1.335–2.840, *P* = 0.001), and composite outcome (HR 1.379, 95% CI 1.096–1.736, *P* = 0.006) compared to the improvement group. The association still remained significant after fully adjusted for both all-cause mortality (HR = 1.899, 95% CI 1.247–2.893, *P* = 0.003) and composite outcome (HR: 1.324, 95% CI: 1.020–1.718, *P* = 0.035).

**Conclusion:** HFmrEF patients are heterogeneous with three different subsets identified, each with different outcomes. Strategies for managing HFmrEF should include previously measured LVEF to allow stratification based on direction changes in LVEF to better optimize treatment.

## Introduction

Heart failure (HF) represents the final common pathway of different cardiac diseases and is a major cause of death among the elderly in many countries (1–4). Currently, risk management and treatment of HF mainly depend on current left ventricular ejection fraction (LVEF) in clinical practice (5, 6). In the latest European Society of Cardiology (ESC) guideline, HF was divided into HF with reduced ejection fraction (HFrEF), HF with mid-range ejection fraction (HFmrEF), and HF with preserved ejection fraction (HFpEF) based on LVEF (7). HFmrEF patients are encountered with an increasing frequency in contemporary HF clinics (8). The latest data show that the prevalence of HFmrEF in hospitalized patients ranged from 13 to 26% (9–11), while the prevalence in outpatients varied from 9 to 21% (12–17). Nevertheless, previous studies mostly focused on HFrEF and HFpEF, with less attention paid to HFmrEF until now (18, 19). Consequently, less is known regarding the clinical characteristics of patients with HFmrEF, and with limited evidence on which to base recommendation for therapy (20).

Indeed, LVEF can be dynamic as the condition of the patient changes. To date, many investigators have been devoting to working on LVEF transition, exploring the incidence, predictors, and associations with outcomes of changes in LVEF in HF patients (21, 22). Some investigators have suggested that HFmrEF patients do not represent a distinct group, but rather represent a heterogeneous group of HFrEF and HFpEF patients, in whom a change in LVEF resulted in their being categorized as a unique subset of HF patients. In their view, HFmrEF represents a transitional state, and can easily progress to HFpEF or HFrEF. However, it must be pointed that transition into the HFmrEF category may also occur by either deterioration or improvement of LVEF. Up to now, there are few studies available describing their characteristics and clinical outcomes. In this study, we examined the association between changes in LVEF and adverse outcomes.

## Materials and Methods

### Study Population

This retrospective cohort study was approved by the institutional review board of the First Affiliated Hospital of the Dalian Medical University. The inclusion criteria were patients admitted for acute decompensated HF at the First Affiliated Hospital of Dalian Medical University between September 1, 2015 and November 31, 2019. The exclusion criterion was a lack of prior echocardiography for comparison. Details of clinical characteristics, comorbidities, drug therapies, laboratory values, and echocardiography findings of the subjects were collected and recorded from Yidu Cloud. All procedures were conducted in accordance with the Declaration of Helsinki. As this was a retrospective research, no informed consents can be obtained.

### Classification of HF Cases

**Figure 1:**
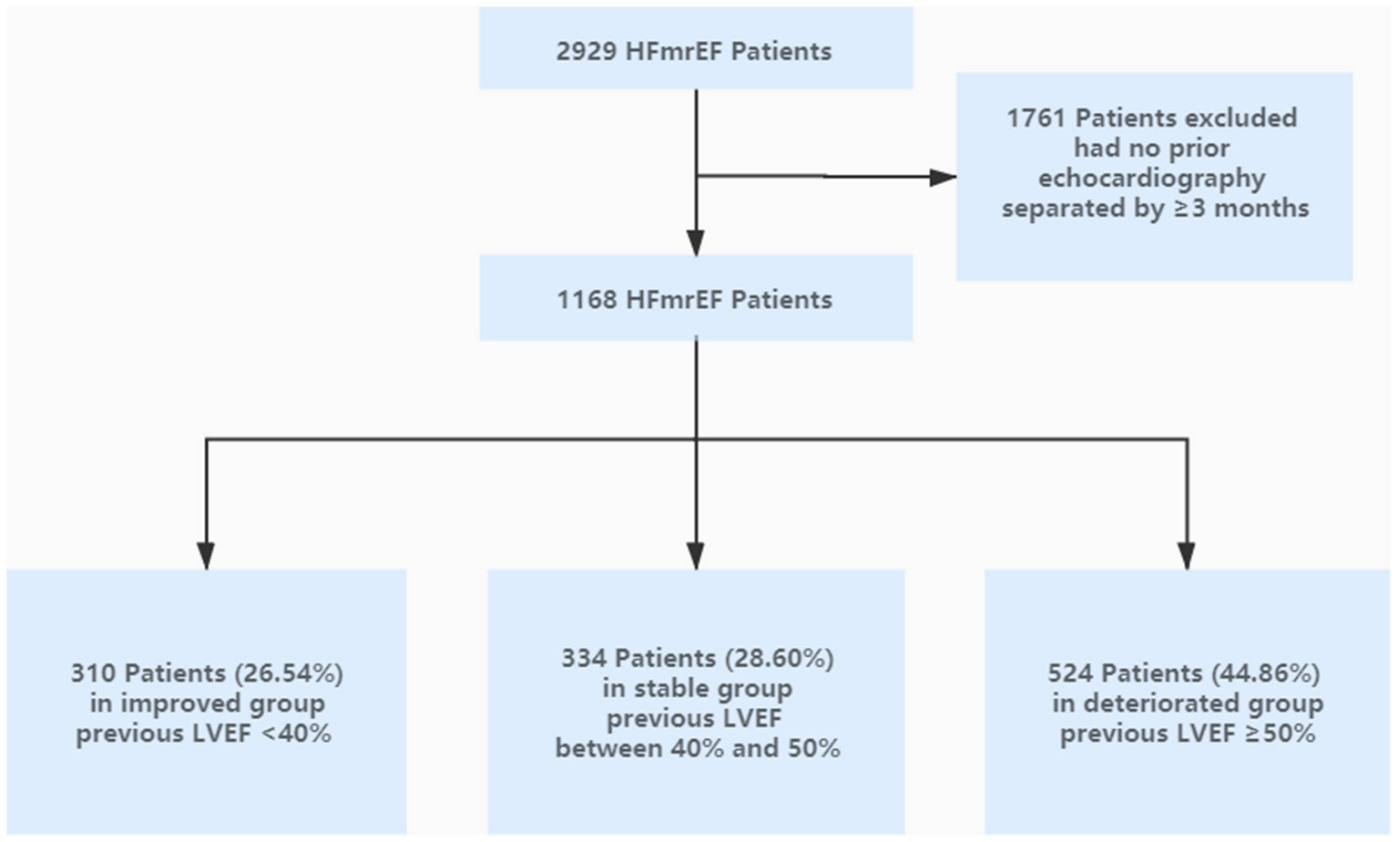
Flow diagram of patient flow.

We classified current HFmrEF patients as having (1) improved group (defined as any previously documented LVEF < 40%), (2) stable group (defined as all previously documented LVEF between 40 and 50%), and (3) deteriorated group (defined as at least one previously documented LVEF ≥ 50%). The study flow chart was shown in Figure 1.

### Clinical Definitions

HF is defined as a clinical syndrome with symptoms and/or signs caused by a structural and/or functional cardiac abnormality and corroborated by elevated natriuretic peptide levels and/or objective evidence of pulmonary or systemic congestion (23). According to echocardiographic data, patients with an EF from 40 to 50% were categorized as HFmrEF.

### Adverse Outcomes

Cardiovascular death, all-cause death, and hospitalization for worsening HF were determined using the Yidu Cloud with complete follow-up through November 30, 2020. The composite endpoint was defined as all-cause hospitalization or all-cause mortality. If these data were unavailable, the status was ascertained by a telephone calling to the patients.

### Statistical Analysis

Statistical analysis was performed using SPSS Statistical Software, Version 22.0 (SPSS Inc., Chicago, IL, USA). Patients' characteristics were summarized with continuous variables expressed as means ± standard deviation and categorical variables presented as frequencies and percentages. Measurement data with a non-normal distribution were expressed as the median (interquartile range). The Kruskal-Wallis test was used for multi-group comparisons, and single-factor ANOVA was used for inter-group comparison. Characteristics were compared across HFmrEF groups using analysis of variance or chi-square tests, as appropriate. Kaplan-Meier analysis was used to describe the cumulative incidence of adverse events, and the long-rank test was used to compare differences.

Univariate and multivariate Cox proportional hazards regression models were used to investigate the risk factors of the endpoints. Covariates selected for multivariate Cox analysis come from either the one with a significance of *P* < 0.05 in the univariate analysis or the one that had been proven to greatly affect the prognosis of HF (Supplementary Tables 1, 2), including age, male, coronary artery disease, hypertension, diabetes mellitus, cerebrovascular disease, ICD, beta-blockers, ACEI/ARB/ARNI, spironolactone, loop diuretics, aspirin, statins, nitrates, hemoglobin, BNP, creatinine, plasma sodium, d-dimer, and time interval. The hazard ratios (HR) and 95% confidence intervals (CI) compare clinical outcomes of cardiovascular death, all-cause death, hospitalization for worsening HF, and composite event of all-cause hospitalization or all-cause mortality for stable group compared with improved group (unadjusted and fully adjusted) and deteriorated group compared with improved group (unadjusted and fully adjusted). All *P*-values represent the significance of the HRs for stable group compared with improved group or deteriorated group compared with improved group. All values were two-tailed, and *P* < 0.05 was considered statistically significant.

## Results

### Demographic and Clinical Characteristics

Of 2,929 patients who had physician-diagnosed HFmrEF at our institution during September 1, 2015 and November 30, 2019, 1,761 patients were excluded due to the lack of availability of an echocardiogram separated by >3 months apart for comparison. A total of 1,168 patients were included (67.04% male, mean age 63.60 ± 12.18 years). The percentages of improved, stable, and deteriorated group were 310 (26.54%), 334 (28.60%), and 524 (44.86%), respectively. The flow chart indicating the inclusion and exclusion criteria was shown in Figure 1.

The baseline characteristics were shown in Table 1. In brief, patients in improved group were younger, had a higher proportion of males, and had a lower frequency of coronary artery disease, cancer, and hypertension compared with those in stable and deteriorated groups. There was no statistical difference in the proportion of NYHA class III–IV between the three groups at the prior echocardiogram. By contrast, improved group showed relative lower prevalence of NYHA class III–IV at the time of inclusion compared to the remaining two groups. Regarding medical therapies, patients in improved group were more likely to take angiotensin converting enzyme inhibitors (ACEI)/angiotensin receptor blockers (ARB)/angiotensin receptor neprilysin inhibitor (ARNI), beta-blockers, spironolactone, loop diuretics, and CRT compared to patients in the remaining two groups. As for laboratory data, the level of white blood cell, hemoglobin, platelet count, uric acid, and BNP in the improved group were significantly higher than other two groups. The average time interval between the two echocardiogram was 16 months. The interval in the deteriorated group was longer than that of the remaining two groups. Prior echocardiography findings showed that patients in improved group had higher left ventricular diameter and left atrial diameter, whereas with lowest value of interventricular septal thickness. Echocardiography findings at the time of inclusion indicated LVEF in all three subgroups fluctuated between 40 and 50, and the value of LVEF in deteriorated group was higher than that of improved group. Moreover, improved group still had the highest left ventricular diameter among the three subgroups; nevertheless, there was no statistical significance across the three groups for the remainder of the parameters.

**Table 1:** Baseline demographics and clinical characteristics of the enrolled heart failure patients stratified by the directional change in LVEF.

**Characteristics**	**All patients (*n* = 1,168)**	**Improved group (*n* = 310)**	**Stable group (*n* = 334)**	**Deteriorated group (*n* = 524)**	***P*-value**
Age (years)	63.60 ± 12.18	60.08 ± 13.08^Ψ^^†^	62.92 ± 12.10^*^^†^	66.11 ± 11.09^*^^Ψ^	<0.0001
Male (*n*, %)	783 (67.04%)	226 (72.90%)^†^	237 (70.96%)^†^	320 (61.07%)^*^^Ψ^	0.0004
Systolic blood pressure (mmHg)	136.2 ± 23.33	133.0 ± 22.22^†^	136.3 ± 23.25	138.0 ± 23.86^*^	0.0118
Diastolic blood pressure (mmHg)	80.46 ± 13.77	81.85 ± 14.11^†^	80.77 ± 13.23	79.43 ± 13.84^*^	0.0437
Heart rates	82.21 ± 22.02	85.76 ± 21.03^†^	82.60 ± 22.87	79.85 ± 21.79^*^	0.0009
Body weight (kg)	73.77 ± 13.26	75.84 ± 15.13^†^	74.38 ± 13.10	72.22 ± 11.97^*^	0.0037
Body mass index (kg/m^2^)	26.15 ± 4.034	26.66 ± 4.1	25.29 ± 3.70	26.27 ± 4.15	0.5814
Prior NYHA class III–IV (*n*, %)	322 (27.56%)	84 (27.10%)	95 (28.44%)	143 (27.29%)	0.9126
NYHA class III–IV at the time of inclusion (*n*, %)	406 (34.76%)	89 (28.70%)^Ψ^^†^	130 (38.92%)^*^	187 (35.68%)^*^	0.0204
**Comorbidities**					
Coronary artery disease (*n*, %)	633 (54.20%)	148 (47.74%)^Ψ^^†^	187 (55.99%)^*^	298 (56.87%)^*^	0.0258
Atrial fibrillation (*n*, %)	310 (26.54%)	67 (21.61%)	93 (27.84%)	150 (28.63%)	0.0699
Cancer (*n*, %)	52 (5.65%)	10 (3.26%)^†^	15 (4.49%)	41 (7.82%)^*^	0.0116
Cerebrovascular disease (*n*, %)	179 (15.33%)	36 (11.61%)	55 (16.47%)	88 (16.79%)	0.1055
Diabetes mellitus (*n*, %)	414 (35.45%)	97 (31.29%)	125 (37.43%)	192 (36.54%)	0.1980
Hypertension (*n*, %)	719 (61.56%)	166 (53.55%)^Ψ^^†^	211 (63.17%)^*^	342 (65.27%)^*^	0.0027
**Therapy**					
ACEI/ARB/ARNI (*n*, %)	652 (55.82%)	187 (60.32%)^†^	197 (58.98%)^†^	268 (51.15%)^*^^Ψ^	0.0139
Aspirin (*n*, %)	683 (58.48%)	176 (56.77%)	209 (62.57%)	298 (56.87%)	0.1982
Beta-blockers (*n*, %)	885 (75.77%)	266 (85.81%)^Ψ^^†^	267 (79.94%)^*^^†^	352 (67.18%)^*^^Ψ^	<0.0001
Digoxin (*n*, %)	154 (13.18%)	62 (20.00%)^†^	36 (10.78%)^*^	56 (10.69%)^*^	0.0002
Loop diuretics (*n*, %)	432 (36.99%)	145 (46.77%)^Ψ^^†^	121 (36.23%)^*^	166 (31.69%)^*^	<0.0001
Nitrates (*n*, %)	438 (37.50%)	103 (33.23%)^Ψ^	144 (43.11%)^*^	191 (36.45%)	0.0280
Spironolactone (*n*, %)	596 (51.03%)	227 (73.23%)^Ψ^^†^	173 (51.80%)^*^^†^	196 (37.40%)^*^^Ψ^	<0.0001
Statins (*n*, %)	763 (65.33%)	197 (63.55%)	235 (70.36%)	331 (63.17%)	0.0726
Warfarin (*n*, %)	226 (19.35%)	55 (17.74%)^†^	52 (15.57%)^†^	119 (22.71%)^*^^Ψ^	0.0252
Pacemaker (*n*, %)	81 (6.93%)	14 (4.52%)	22 (6.59%)	45 (8.59%)	0.0784
ICD (*n*, %)	18 (1.54%)	8 (2.58%)	5 (1.50%)	5 (0.95%)	0.1825
CRT (*n*, %)	22 (1.88%)	14 (4.52%)^Ψ^^†^	5 (1.50%)^*^	3 (0.57%)^*^	0.0002
**Laboratory values**					
White blood cell (10∧9/L)	7.655 ± 3.135	8.061 ± 3.386^†^	7.595 ± 3.007	7.452 ± 3.042^*^	0.0231
Hemoglobin (g/L)	136.9 ± 21.64	141.1 ± 21.85^Ψ^^†^	136.9 ± 20.66^*^	134.4 ± 21.77^*^	<0.0001
Platelet (10∧9/L)	208.7 ± 66.64	222.4 ± 80.98^Ψ^^†^	202.0 ± 59.32^*^	205.0 ± 60.34^*^	0.0001
Creatinine (μmol/L)	76.00 (62.00, 97.00)	79 (64.25, 99.00)	76.00 (63.00, 98.00)	74.00 (61.00, 95.00)	0.6160
UA (μmol/L)	409.4 ± 138.0	440.9 ± 161.1^Ψ^^†^	412.5 ± 131.9^*^	390.3 ± 124.5^*^	<0.0001
Na^+^ (mmol/L)	141.7 ± 3.130	141.6 ± 3.169	141.6 ± 3.021	141.7 ± 3.179	0.7728
Glu (mmol/L)	6.351 ± 2.614	6.370 ± 2.853	6.373 ± 2.489	6.326 ± 2.560	0.9619
D-dimer (μg/L)	420 (210.0, 970.0)	410 (210.0, 970.0)	410 (190.0 880.0)	455.0 (230.0, 1,025)	0.2193
BNP level (ng/L)	317.5 (119.9, 779.4)	506.7 (183.5, 1,168)^†^	337.4 (127.0, 922.1)^†^	231.2 (90.40, 517.9)^*^^Ψ^	<0.0001
**Echocardiography parameters**					
Time interval (months)	16.00 (7.250, 29.00)	12.00 (6.000, 26.00)^†^	13.50 (7.000, 27.00)^†^	19.00 (10.00, 31.00)^*^^Ψ^	<0.0001
**Prior echocardiography findings**					
Left ventricular ejection fraction (%)	46.26 ± 10.57	32.41 ± 5.626^Ψ^^†^	43.61 ± 2.711^*^^†^	56.16 ± 3.088^*^^Ψ^	<0.0001
Left ventricular diameter (mm)	53.52 ± 7.875	59.69 ± 7.213^Ψ^^†^	54.22 ± 6.706^*^^†^	49.61 ± 6.466^*^^Ψ^	<0.0001
Left atrial diameter (mm)	42.44 ± 7.225	44.02 ± 6.313^Ψ^^†^	42.64 ± 6.711^*^^†^	41.44 ± 7.838^*^^Ψ^	<0.0001
Interventricular septal thickness (mm)	10.68 ± 1.914	10.38 ± 1.661^Ψ^^†^	10.80 ± 1.951^*^	10.78 ± 2.007^*^	0.0103
E/e′	13.02 ± 5.621	13.46 ± 5.383	13.47 ± 6.075	12.38 ± 5.372	0.0534
**Echocardiography findings at the time of inclusion**					
Left ventricular ejection fraction (%)	43.75 ± 2.875	43.35 ± 2.874^†^	43.70 ± 2.757	44.02 ± 2.925^*^	0.0047
Left ventricular diameter (mm)	53.92 ± 6.604	56.24 ± 6.259^Ψ^^†^	54.78 ± 6.759^*^^†^	51.84 ± 6.076^*^^Ψ^	<0.0001
Left atrial diameter (mm)	42.85 ± 6.782	42.28 ± 6.630	43.52 ± 6.433	42.77 ± 7.079	0.0682
Interventricular septal thickness (mm)	10.76 ± 1.998	10.55 ± 1.871	10.73 ± 2.153	10.90 ± 1.960	0.0568
E/e′	13.07 ± 5.628	12.19 ± 5.645	13.18 ± 5.344	13.49 ± 5.770	0.0668

*NYHA, New York Heart Association; ACEI, angiotensin-converting enzyme inhibitor; ARB, angiotensin II receptor blocker; ARNI, angiotensin receptor neprilysin inhibitor; ICD, implantable cardioverter defibrillator; CRT, cardiac resynchronization therapy; BNP, B-type natriuretic peptide; Glu, glucose; UA, uric acid; E/e′, mitral Doppler early velocity/mitral annular early velocity*.

* *is compared with Improved group P < 0.05*,

Ψ *is compared with Stable group P < 0.05*,

† *is compared with Deteriorated group P < 0.05*.

### Clinical Outcomes

**Figure 2:**
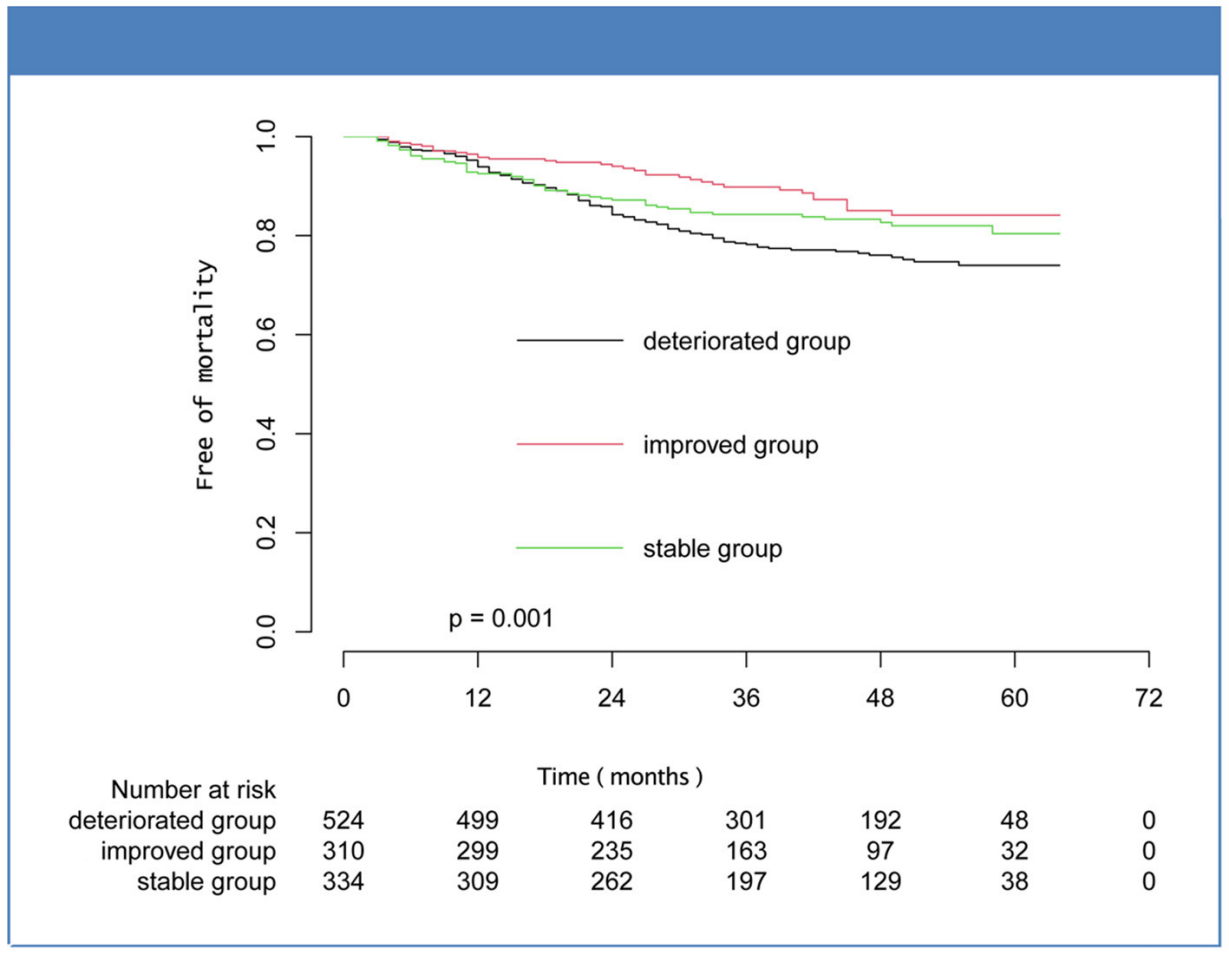
Kaplan-Meier curves for mortality for the subsets of heart failure with midrange ejection fraction.

**Figure 3:**
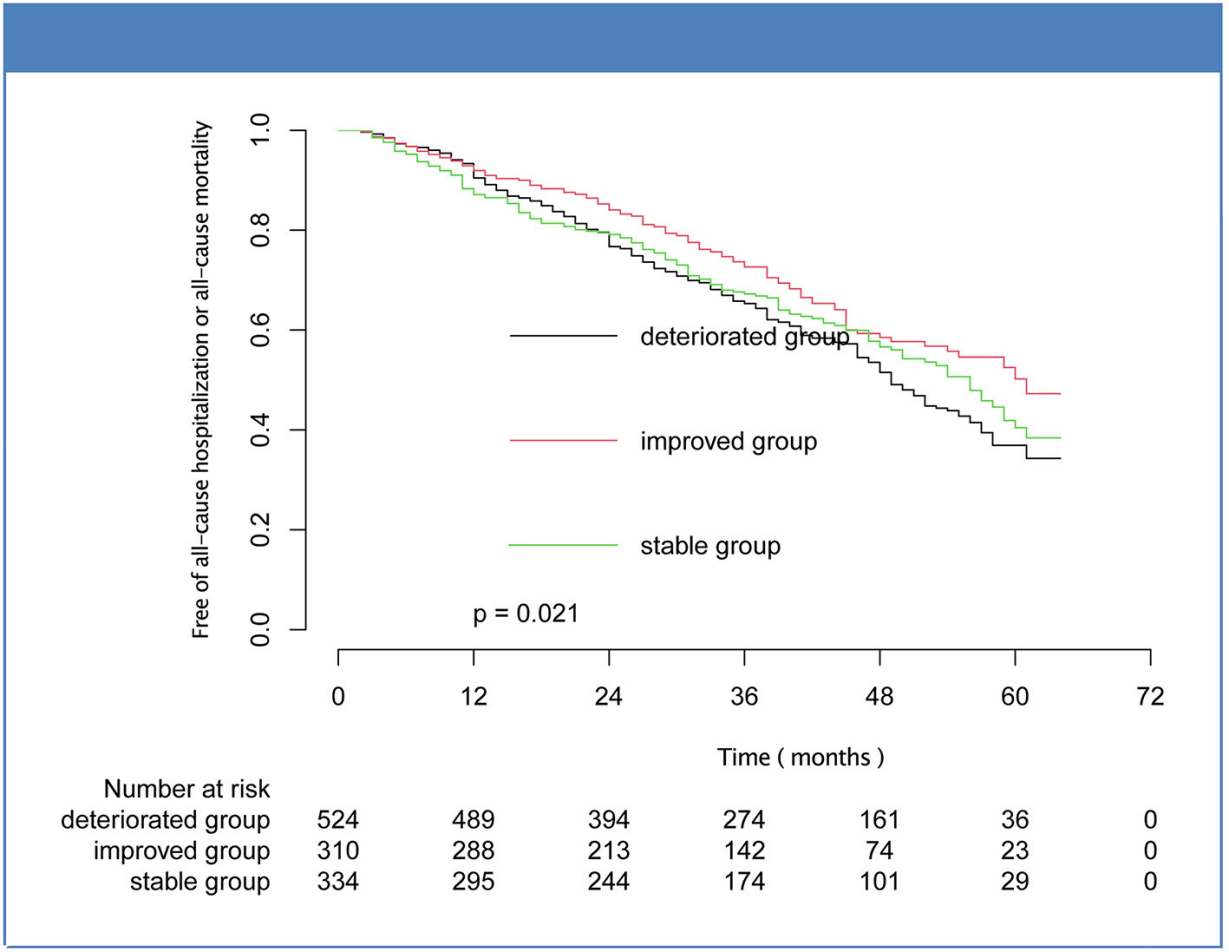
Kaplan-Meier curves for composite outcome of mortality or hospitalization for the subsets of heart failure with midrange ejection fraction.

Over a median follow-up of 40.00 [25.00–53.00] months, there were 208 patients (17.81%) deaths, and the percentages of improved, stable, and deteriorated group were 35 (11.29%), 55 (16.47%), and 118 (22.52%), respectively. Five hundred patients met the composite endpoint (42.81%), and the number were 102 (32.90%), 145 (43.41%), and 253 (48.28%) for the improved, stable, and deteriorated groups, respectively. Kaplan-Meier analysis showed that the mortality and composite outcome in improved group was significantly lower than that in stable and deteriorated groups (Figures 2, 3). However, there was no statistical difference in the rates of cardiovascular death and hospitalization for worsening HF among the three subsets (Supplementary Figures 1, 2).

**Figure 4:**
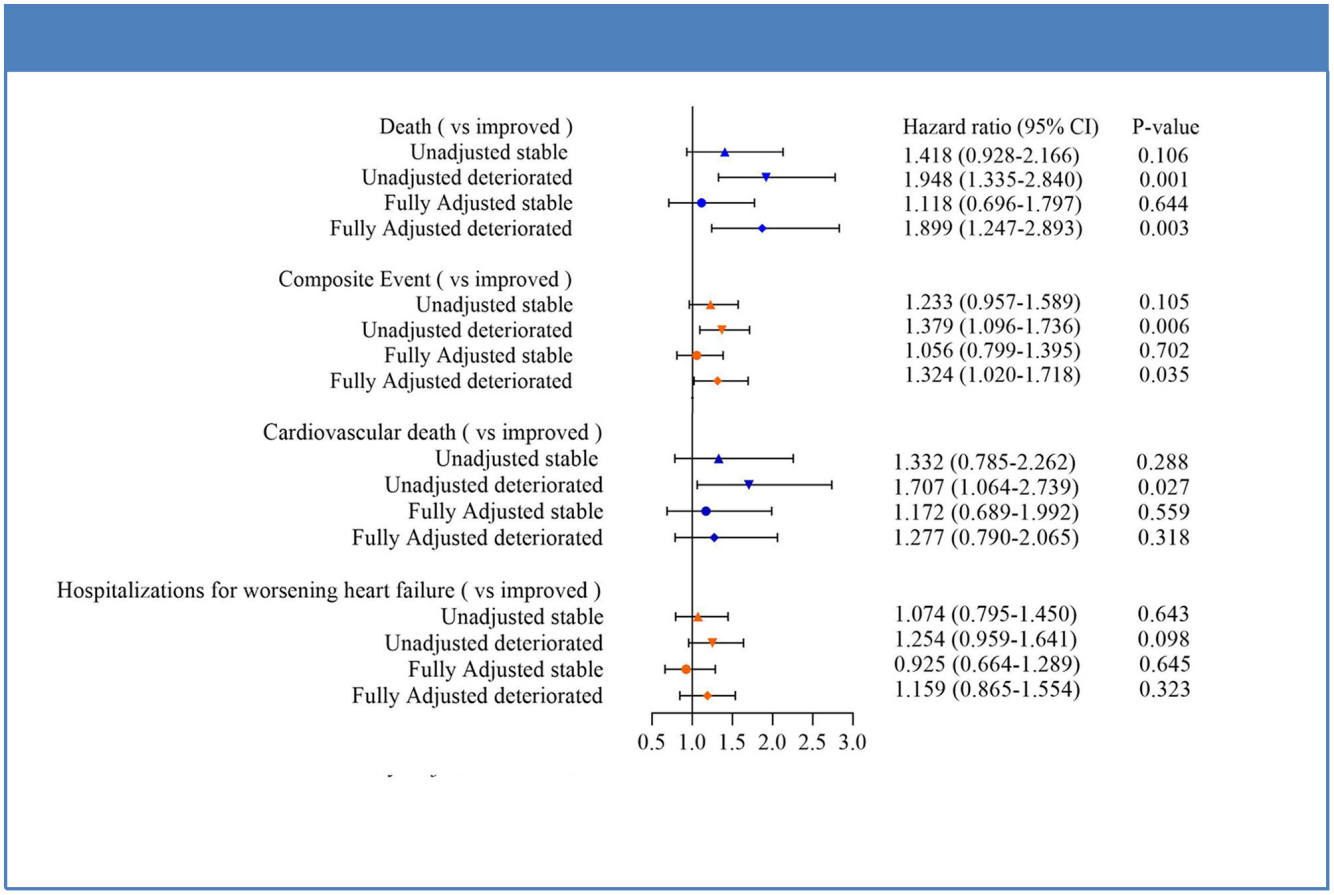
Forest plot of clinical outcomes in HFmrEF subgroups.

Cox regression analysis indicated that the deteriorated group showed a significantly higher risk of composite endpoint compared with patients in improved group (HR 1.379, 95% CI 1.096–1.736, *P* = 0.006). This difference was mainly due to trends toward increased risk of all-cause mortality (HR 1.948, 95% CI 1.335–2.840, *P* = 0.001). The association remained significant after adjustment for potential confounders for both mortality (HR = 1.899, 95% CI 1.247–2.893, *P* = 0.003) and composite outcome (HR: 1.324, 95% CI: 1.020–1.718, *P* = 0.035). Moreover, compared to improved group, deteriorated group also experienced a 1.71-fold increase in risk of cardiovascular death (HR: 1.707, 95% CI: 1.064–2.739, *P* = 0.027), albeit not reaching statistical significance in fully adjusted analysis. As with outcomes for hospitalization for worsening HF, HRs between the three subgroups did not show statistical differences. In addition, no significant differences in outcomes between patients in the improved and stable groups were seen for any of the endpoints in either unadjusted or fully adjusted analysis. The results were shown in Figure 4.

## Discussion

This study demonstrated that HFmrEF patients were a heterogeneous group of patients comprised of at least three different subsets. Additionally, the characteristics and clinical outcomes of HFmrEF patients among subgroups defined by the prior directional changes in LVEF are significantly different.

Risk stratification in HF is an important clinical problem (24, 25). Previous studies have elucidated that the demographics of patients with HFmrEF lied in between those of HFpEF and HFrEF patients, but in general were more similar to HFpEF patients, with a heavier burden of hypertension and atrial fibrillation/flutter (10, 12, 13, 26). Nevertheless, HFmrEF also resembled HFrEF showing a higher burden of ischemic heart disease (9, 27–29). In our study, we found that the HFmrEF cohort suffered from a heavy burden of comorbidities, such as hypertension (61.56%), coronary heart disease (54.20%), and atrial fibrillation/flutter (26.54%). Our research also indicated that the characteristics of patients within HFmrEF subgroup were significantly different from the HFrEF and HFpEF subgroups. For example, patients in the deteriorated group were older, more female, and more likely to have hypertension, which were features consistent with HFpEF. By contrast, the deteriorated cohort had higher prevalence of coronary artery disease, which was in keeping with a HFrEF phenotype.

Regarding treatment, previous literatures suggested that HFmrEF patients received a mixture of medications indicated for both HFrEF and HFpEF patients (30, 31). Indeed, our study found that HFmrEF patients were prescribed medications recommended for HFrEF (digoxin, ACEI or ARB) as well as for HFpEF (calcium channel blockers). In our cohort, more than 50% patients received the traditional first-line agents of beta-blockers, ACEI/ARB, and aldosterone antagonist. Moreover, we found that patients in the improved group were more likely to take beta-blockers, ACEI/ARB/ARNI, spironolactone, and CRT than patients in the remaining two groups. The reason may be that neurohormonal blocking agents were only recommended for the patients with HFrEF but not HFpEF in HF management guidelines. Overall, these discrepancies underscored the considerable heterogeneity between patients in the HFmrEF population.

Notably, prior studies illustrated that the prognosis of HFmrEF patients was distinct from those of HFrEF and HFpEF. A 5-year follow-up of mortality showed that all-cause mortality in HFmrEF was higher than the rate of HFpEF patients, but lower than that of HFrEF patients (32, 33). However, HFmrEF mortality at 1 year after discharge was similar to that of HFpEF (10, 34, 35). The findings from four community-based longitudinal cohorts showed that age was an important clinical predictor of new onset HFmrEF (27). Meanwhile, a latest separate study demonstrated age ≥80 years was associated with a higher risk of mortality within 1 year following discharge in the HFmrEF group compared with other HF types (35). In this study, our results also identified age as an independent risk factor for both mortality and composite outcome. Moreover, we found the adverse events of patients with HFmrEF varied considerably between subgroups and the clinical course was closely associated with the directional changes in LVEF that brought them into the mid-range. Unsurprisingly, patients in deteriorated group had a worse prognosis compared to other HFmrEF phenotypes, with a remarkably increased risk of a median follow-up of 40.00 months mortality and hospitalization, indicating the urgent need for careful follow-up of this group. The unfavorable outcomes may be related to a large reduction of LVEF and a substantial increase in LV diameter in deteriorated group. The adverse alternations in cardiac structure and function are most likely due to the lower usage of guideline-directed medical therapy and the relatively high prevalence of coronary artery disease, as coronary artery disease was always associated with higher risk of mortality and worsening LVEF. In the large Improve Heart Failure Therapies in the Outpatient Setting registry, patients without prior myocardial infarction and non-ischemic HF etiology were both associated with a >10% improvement in LVEF (36).

These findings suggested that for HFmrEF patients, previous changes in the direction of LVEF may provide important prognostic value, and clinicians should consider previous changes in LVEF when devising treatment plans.

### Limitations

Nevertheless, we must note that this study still has several limitations. Firstly, considering the single-center nature of our study, the findings may not be generalizable to other settings. Secondly, the interval between the prior echocardiogram and the inclusion to the study was not exactly the same. Patients with echocardiography assessments within a short time period might have been less likely to exhibit a change in EF category. Although multivariate Cox regression models were applied to adjust for the interval between echocardiography assessments, residual confounding might have been a limitation. Thirdly, we can only obtain the medical record of patients hospitalized at our center, and we have no way of confirming when HF was first diagnosed, as this might have taken place at other hospitals. Thus, in this study, not every patient's echocardiogram time relative to initial HF diagnosis can be clearly recorded. Lastly, clinical outcomes were ascertained mainly depending on a telephone calling to the patients. Therefore, only a small number of patients in this cohort underwent the last follow-up echocardiography. In the near future, a large prospective cohort or a randomized-controlled study is necessary to understand the characteristics and evaluate the effects of drugs in HFmrEF population.

### Conclusions

In conclusion, differences in the prevalence of risk factors and underlying etiology may generate different effects on LVEF transition, and thus different outcomes. The condition of HFpEF to HFmrEF is a dangerous and complex pathological process, which always implied worse clinical outcomes. These findings would remind clinicians to pay more attention to previous echocardiography results in HFmrEF patients, and to consider the impact of direction changes in LVEF on the prognosis of patients when planning management strategies.

## Data Availability Statement

The raw data supporting the conclusions of this article will be made available by the authors, without undue reservation.

## Ethics Statement

The studies involving human participants were reviewed and approved by the Institutional Review Board of Dalian Medical University. Written informed consent for participation was not required for this study in accordance with the national legislation and the institutional requirements.

## Author Contributions

XZ and YS were responsible for collecting clinical data and writing the paper. YZ and FC assisted YS in collecting data and conducting telephone follow-up. HH helped YZ with the follow-up. SS and SZ were responsible for the statistical analysis. YL and GT were responsible for revising the paper and determining the research direction. All authors were involved in the drafting or revision of the manuscript.

## Conflict of Interest

The authors declare that the research was conducted in the absence of any commercial or financial relationships that could be construed as a potential conflict of interest.

## Publisher's Note

All claims expressed in this article are solely those of the authors and do not necessarily represent those of their affiliated organizations, or those of the publisher, the editors and the reviewers. Any product that may be evaluated in this article, or claim that may be made by its manufacturer, is not guaranteed or endorsed by the publisher.
