# Supplementary material for: Heart Failure With Midrange Ejection Fraction: Prior Left Ventricular Ejection Fraction and Prognosis
Source: Front Cardiovasc Med. 2021 Aug 2;8:697221. doi: 10.3389/fcvm.2021.697221 (PMC8364975; doi:10.3389/fcvm.2021.697221)

**Supplementary table 1.** Cox regression to identify significant predictors of all-cause mortality in HFmrEF subgroups.

| **Characteristics** | **Univariate analysis** | | | **Multivariate analysis** | | |
| --- | --- | --- | --- | --- | --- | --- |
|  | **HR** | **95%CI** | **P value** | **HR** | **95%CI** | **P value** |
| Age | 1.063 | 1.049-1.078 | 0.000 | 1.060 | 1.043-1.0477 | 0.000 |
| Male | 0.729 | 0.552-0.963 | 0.026 | 0.850 | 0.624-1.160 | 0.306 |
| Coronary artery disease | 1.502 | 1.133-1.992 | 0.005 | 0.955 | 0.683-1.335 | 0.788 |
| Hypertension | 1.579 | 1.168-2.135 | 0.003 | 0.931 | 0.640-1.355 | 0.709 |
| Diabetes mellitus | 1.727 | 1.315-2.266 | 0.000 | 1.671 | 1.241-2.249 | 0.001 |
| Cerebrovascular disease | 1.774 | 1.291-2.439 | 0.000 | 1.320 | 0.938-1.859 | 0.112 |
| Beta-blockers | 0.645 | 0.481-0.863 | 0.003 | 0.877 | 0.628-1.224 | 0.439 |
| Spironolactone | 0.736 | 0.559-0.968 | 0.029 | 1.067 | 0.773-1.473 | 0.693 |
| Nitrates | 1.478 | 1.126-1.941 | 0.005 | 1.171 | 0.867-1.581 | 0.303 |
| Time interval | 0.959 | 0.946-0.970 | 0.000 | 0.955 | 0.942-0.968 | 0.000 |
| Hemoglobin | 0.985 | 0.980-0.990 | 0.000 | 0.990 | 0.984-0.997 | 0.003 |
| BNP | 1.000 | 1.000-1.000 | 0.022 | 1.000 | 1.000-1.000 | 0.001 |
| Creatinine | 1.001 | 1.000-1.003 | 0.057 | 1.001 | 0.999-1.003 | 0.280 |
| D-dimer | 1.000 | 1.000-1.000 | 0.036 | 1.000 | 1.000-1.000 | 0.127 |

Abbreviations: BNP, B-type natriuretic peptide.

**Supplementary table 2.** Cox regression to identify significant predictors of all-cause mortality or hospitalization in HFmrEF subgroups.

| **Characteristics** | **Univariate analysis** | | | **Multivariate analysis** | | |
| --- | --- | --- | --- | --- | --- | --- |
|  | **HR** | **95%CI** | **P value** | **HR** | **95%CI** | **P value** |
| Age | 1.027 | 1.019-1.035 | 0.000 | 1.020 | 1.011-1.029 | 0.000 |
| Male | 0.839 | 0.698-1.008 | 0.061 | 0.801 | 0.649-0.989 | 0.039 |
| Coronary artery disease | 1.322 | 1.105-1.581 | 0.002 | 0.931 | 0.743-1.167 | 0.535 |
| Hypertension | 1.442 | 1.193-1.743 | 0.000 | 1.089 | 0.859-1.380 | 0.481 |
| Diabetes mellitus | 1.461 | 1.224-1.744 | 0.000 | 1.156 | 0.927-1.441 | 0.197 |
| Cerebrovascular disease | 1.514 | 1.219-1.881 | 0.000 | 1.326 | 1.049-1.676 | 0.018 |
| ICD | 0.294 | 0.095-0.916 | 0.035 | 0.473 | 0.151-1.476 | 0.197 |
| Beta-blockers | 0.770 | 0.632-0.937 | 0.009 | 0.881 | 0.709-1.094 | 0.253 |
| ACEI/ARB/ARNI | 0.834 | 0.699-0.994 | 0.043 | 0.921 | 0.755-1.123 | 0.414 |
| Spironolactone | 0.656 | 0.548-0.784 | 0.000 | 0.719 | 0.590-0.876 | 0.001 |
| Loop diuretics | 0.767 | 0.635-0.926 | 0.006 | 0.855 | 0.676-1.081 | 0.189 |
| Aspirin | 1.255 | 1.045-1.508 | 0.015 | 0.977 | 0.748-1.277 | 0.865 |
| Statins | 1.266 | 1.045-1.533 | 0.016 | 0.884 | 0.704-1.111 | 0.290 |
| Nitrates | 1.283 | 1.075-1.531 | 0.006 | 1.138 | 0.937-1.381 | 0.191 |
| Time interval | 0.971 | 0.965-0.978 | 0.000 | 0.955 | 0.942-0.968 | 0.000 |
| Hemoglobin | 0.989 | 0.986-0.993 | 0.000 | 0.995 | 0.991-1.000 | 0.060 |
| BNP | 1.000 | 1.000-1.000 | 0.061 | 1.000 | 1.000-1.000 | 0.148 |
| Creatinine | 1.002 | 1.001-1.003 | 0.000 | 1.002 | 1.001-1.003 | 0.000 |
| Plasma sodium | 0.994 | 0.917-0.972 | 0.000 | 0.950 | 0.922-0.978 | 0.001 |

Abbreviations: ICD, implantable cardioverter defibrillator; ACEI, angiotensin-converting enzyme inhibitor; ARB, angiotensin II receptor blocker; ARNI, angiotensin receptor neprilysin inhibitor; BNP, B-type natriuretic peptide.

**Supplementary figure 1.** Kaplan-Meier curves for cardiovascular death for the subsets of heart failure with mid-range ejection fraction


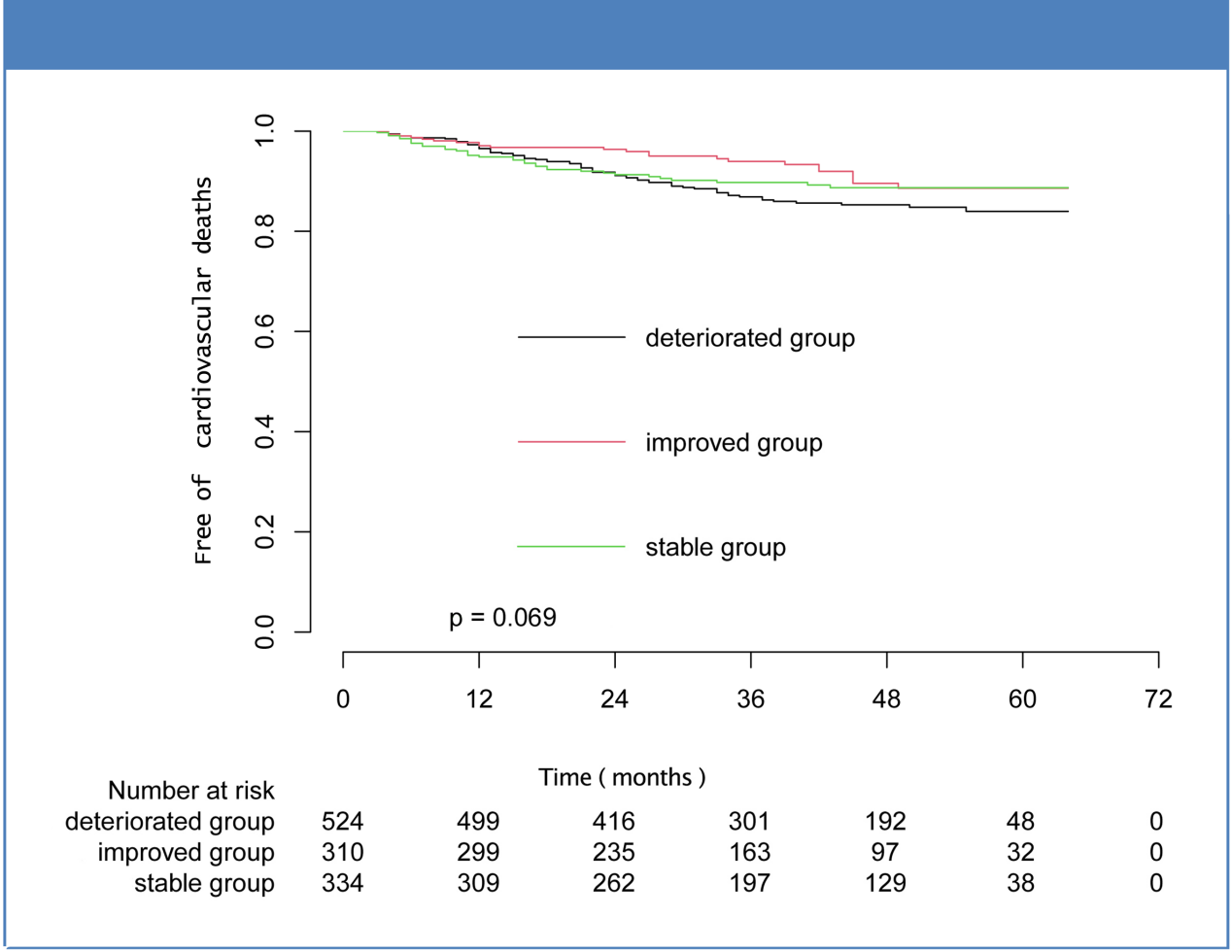


**Supplementary figure 2.** Kaplan-Meier curves for hospitalization for worsening heart failure for the subsets of heart failure with mid-range ejection fraction


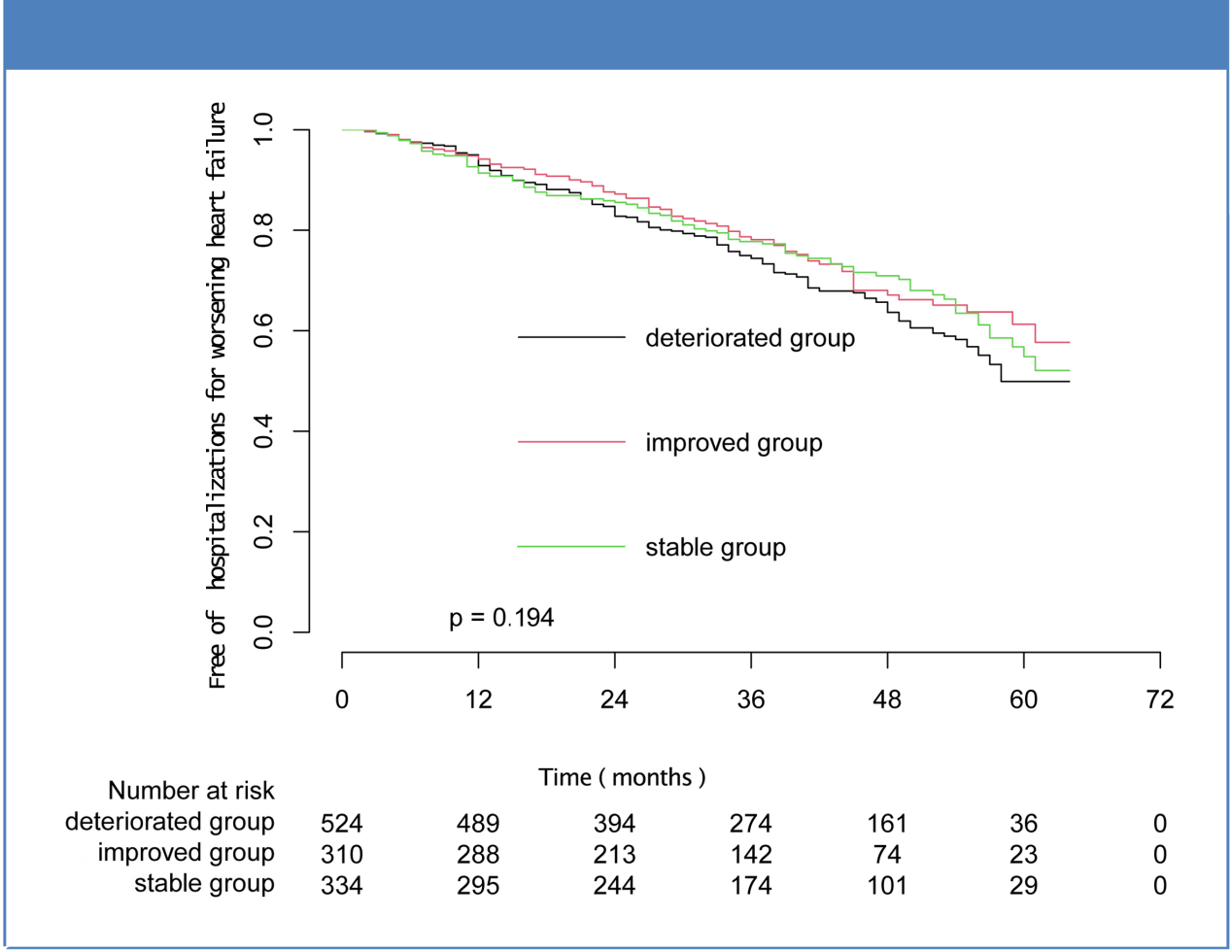

Supplement: Supplementary file 1 [file Data_Sheet_1.docx]
